# Supplementary material for: PI(3,4,5)P3 allosteric regulation of repressor activator protein 1 controls antigenic variation in trypanosomes
Source: eLife. 2023 Nov 29;12:RP89331. doi: 10.7554/eLife.89331 (PMC10686619; doi:10.7554/eLife.89331)
Supplement: Supplementary file 5. — RNA-seq in cells expressing WT or Mut (D360A/N362A) PIP5Pase, and ChIP-seq of RAP1-HA in cells expressing WT or Mut PIP5Pase. [file elife-89331-supp5.docx]

| **Experiment** | **Treatment** | **Replicates** | **Reads mean length** | **Sequences (bp)** |  |
| --- | --- | --- | --- | --- | --- |
| RNA-seq | WT PIP5Pase | Bio1 | 668.919 | 2,072,167,984 |  |
|  |  |  |  |  |  |
|  |  | Bio2 | 2547.51 | 1,990,123,451 |  |
|  |  |  |  |  |  |
|  |  | Bio3 | 400.328 | 317,681,697 |  |
|  |  |  |  |  |  |
|  | Mut PIP5Pase | Bio1 | 639.227 | 1,697,997,595 |  |
|  |  |  |  |  |  |
|  |  | Bio2 | 492.199 | 320,814,615 |  |
|  |  |  |  |  |  |
|  |  | Bio3 | 237.398 | 533,192,072 |  |
|  |  |  |  |  |  |
| ChIP-seq | RAP1-HA in WT PIP5Pase | Bio1_Input | 323.63 | 465,606,176 |  |
|  |  | Bio1_ChIP | 369.573 | 588,132,165 |  |
|  |  | Bio2_Input | 722.822 | 879,935,255 |  |
|  |  | Bio2_ChIP | 587.867 | 257,141,699 |  |
|  |  | Bio3_Input | 749.543 | 698,570,934 |  |
|  |  | Bio3_ChIP | 285.618 | 678,626,652 |  |
|  | RAP1-HA in Mut PIP5Pase | Bio1_Input | 570.146 | 295,541,382 |  |
|  |  | Bio1_ChIP | 282.222 | 497,713,577 |  |
|  |  | Bio2_Input | 597.497 | 202,006,025 |  |
|  |  | Bio2_ChIP | 322.475 | 577,598,596 |  |
|  |  | Bio3_Input | 491.412 | 196,564,842 |  |
|  |  | Bio3_ChIP | 480.533 | 205,665,425 |  |
